# Distinct response of the hepatic transcriptome to Aflatoxin B_1_ induced hepatocellular carcinogenesis and resistance in rats

**DOI:** 10.1038/srep31898

**Published:** 2016-08-22

**Authors:** Jiejun Shi, Jiangtu He, Jing Lin, Xin Sun, Fenyong Sun, Chao Ou, Cizhong Jiang

**Affiliations:** 1 Department of Clinical Laboratory Medicine, Shanghai Tenth People’s Hospital of Tongji University, Shanghai, China; 2 Shanghai Key Laboratory of Signaling and Disease Research, the School of Life Sciences and Technology, Tongji University, Shanghai, China; 3 Department of Clinical Laboratory, the Affiliated Tumor Hospital of Guangxi Medical University, Nanning, Guangxi Province, China

## Abstract

Aflatoxin is a natural potent carcinogen and a major cause of liver cancer. However, the molecular mechanisms of hepatocellular carcinogenesis remain largely unexplored. In this study, we profiled global gene expression in liver tissues of rats that developed hepatocellular carcinoma (HCC) from aflatoxin B_1_ (AFB_1_) administration and those that were AFB_1_-resistant, as well as rats without AFB_1_ exposure as a control. AFB_1_ exposure resulted in extensive perturbation in gene expression with different functions in HCC and AFB_1_ resistance (AR) samples. The differentially expressed genes (DEGs) in HCC sample were enriched for cell proliferation, cell adhesion and vasculature development that largely contribute to carcinogenesis. Anti-apoptosis genes were up-regulated in HCC sample whereas apoptosis-induction genes were up-regulated in AR sample. AFB_1_ exposure also caused extensive alteration in expression level of lncRNAs. Among all the 4511 annotated lncRNAs, half of them were highly expressed only in HCC sample and up-regulated a group of protein-coding genes with cancer-related functions: apoptosis regulation, DNA repair, and cell cycle. Intriguingly, these genes were down-regulated by lncRNAs highly expressed in AR sample. Collectively, apoptosis is the critical biological process for carcinogenesis in response to AFB_1_ exposure through changes in expression level of both protein-coding and lncRNA genes.

Aflatoxins are potent mycotoxins produced by the fungi *Aspergillus flavus* and *Aspergillus parasiticus*, and contaminate a variety of food commodities, including rice, maize, and groundnuts. Dietary exposure to aflatoxins highly contributes to hepatocellular carcinoma (HCC)^1^,^2^. HCC is one of the leading causes of cancer death worldwide. Therefore, food contamination with aflatoxins is detrimental to human health, especially to the people in tropical and subtropical regions with high level of dietary exposure to aflatoxins because of the high temperature and humidity.

Aflatoxins are a group of secondary metabolites produced by molds grown on crops. There are four major aflatoxins known as B_1_, B_2_, G_1_, and G_2_^3^. Among them, aflatoxin B_1_ (AFB_1_) is the most toxic mycotoxin to which humans are exposed^4^,^5^ and the most potent naturally occurring chemical liver carcinogen known^6^. Previous studies have shown that AFB_1_ is a potent mutagen and hepatocarinogen^7^,^8^. AFB_1_ is metabolized into the reactive oxygen species (ROS): aflatoxin-8,9-expoxide^9^. This ROS can react with proteins and DNA to form adducts and cause acute toxicity (aflatoxicosis) and lesions, respectively^10^,^11^. Failure in repair of the lesion may lead to DNA mutation, preferentially G to T transversions. A couple of studies identified the mutation (AGG to AGT, R249S) at codon 249 in the *TP53* tumor suppressor gene, specific for exposure to aflatoxin^12^,^13^. Recent studies have confirmed that this hotspot in *TP53* is a preferential site for AFB_1_ adduct formation^14^. Therefore formation of AFB_1_-DNA adducts over time increases the risk of HCC.

An *in vivo* study also showed that AFB_1_ induced lipid peroxidation in the liver of Fischer 344 rats^5^. Lipid peroxidation is the major form of the cellular oxidative damages that is initiated by hydroxyl-free radical^15^. The reaction of lipid peroxidation retracts a hydrogen atom from unsaturated fatty acids of membrane phospholipids, destructs cellular membrane, and results in other cytotoxic responses^16^.

Additionally, aflatoxins appear to have synergistic effects on hepatitis B virus (HBV)- and hepatitis C virus (HCV)-induced liver cancer. The missense mutation R249S in *TP53* is common in HCC of people who live in regions where both HBV carriage and aflatoxin exposure are highly prevalent, but not in regions where HBV is prevalent alone^17^,^18^. Other studies also showed that aflatoxin exposure can accelerate carcinogenesis of HBV or HCV infection by introducing mutations^19^,^20^.

The role of aflatoxin in disturbing gene expression has attracted much attention. AFB_1_-DNA or -RNA adducts can block transcription and translation^21^,^22^,^23^. RNA-sequencing analysis of hepatic gene expression in growing barrows administered AFB_1_ found changes in expression of apoptosis genes, such as cyclin-dependent kinas inhibitor 1A, zinc finger matrin type 3, kininogen 1, and pin-1 oncogene^24^. Another hepatic transcriptomic study in domestic turkey showed that AFB_1_ exposure affected expression of genes related to cancer, apoptosis, cell cycle, or lipid regulation^25^. Hepatic RNA-seq profiling of AFB_1_-treated rats revealed that differentially expressed genes were enriched in the pathways of cell cycle, extracellular matrix, cell differentiation network, and E2f1-related pathways^26^. MicroRNAs also play important roles in tumorigenesis. Deep sequencing analysis of microRNA in rat livers administered AFB_1_ found abnormal expression in cancer-related microRNAs whose target genes were involved in cancer-related pathways^27^.

In this study, we applied high-throughput RNA-seq technology to investigate the molecular mechanisms of AFB_1_ carcinogenesis and resistance in rat liver tissues. AFB_1_ exposure caused extensive change in expression of both protein-coding and lncRNA genes. Moreover, almost half lncRNAs were positively correlated with a group of protein-coding genes in hepatocellular carcinogenesis from AFB_1_ exposure. These genes were negatively correlated with a small number of lncRNAs highly expressed in AR sample. Gene Ontology (GO) analysis of these genes identified enrichment of regulation of apoptosis, DNA repair, and cell cycle. In contrast, lncRNAs regulated protein-coding genes in an intricate fashion. Further analysis revealed that anti-apoptosis genes were up-regulated in hepatocellular carcinogenesis, whereas apoptosis-induction genes were up-regulated in AFB_1_ resistance. Our study sheds new light on the coordinated expression change of protein-coding and lncRNA genes in hepatocellular carcinogenesis and AFB_1_ resistance.

## Results

### Different disturbance in gene expression in AFB_1_ carcinogenesis and resistance

To gain insights into the molecular mechanisms by which AFB_1_ exposure induces HCC, whereas some rats showed AFB_1_ resistance, we profiled gene expression in three groups of rats: the control group without AFB_1_ exposure (denoted as Ctrl hereinafter), the AFB_1_ hepatocellular carcinoma group (denoted as HCC hereinafter), and the AFB_1_ resistance group (denoted as AR hereinafter). Both HCC and AR group of rats were administered AFB_1_ (see Methods for details). HCC group developed HCC and AR group did not. Ctrl group did not develop HCC, either. RNA-sequencing generated approximately 30 million read pairs with read length 90 nucleotides for each sample (Supplementary Table S1). 87% of read pairs were uniquely concordantly mapped to annotated genes. This suggests high quality of the sequencing results.

**Figure 1:**
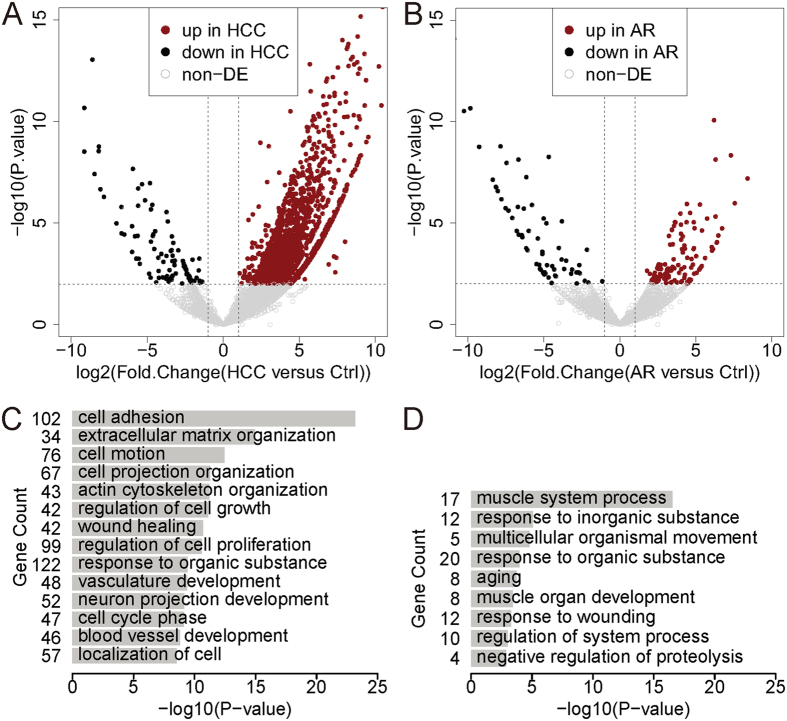
Extensive disturbance in gene expression in AFB_1_ hepatocellular carcinogenesis and resistance. Volcano plots show differentially expressed genes (DEGs) in the hepatocellular carcinoma (HCC) sample (**A**) and the AFB_1_ resistance (AR) sample (**B**) compared to the control sample. Bar plots show the biological process GO terms for which the DEGs in the HCC sample (**C**) and the AR (**D**) sample are enriched.

We next identified differentially expressed genes (DEGs) by comparing two samples. There are 1,452 DEGs between the HCC sample and the Ctrl sample, and 141 DEGs between the AR sample and the Ctrl sample (Fig. 1A,B). This indicates that AFB_1_ exposure resulted in a more extensive disturbance in gene expression in the HCC sample than in the AR sample. Genes related to AFB_1_ metabolism or detoxicity, e.g. cytochrome P450 and glutathione transferases^28^, also presented in the DEGs lists, and their up-regulation in AR sample was validated by qPCR (Supplementary Fig. S1). The Gene Ontology (GO) analysis found DEGs between the HCC sample and the Ctrl sample were enriched for cell adhesion, extracellular matrix organization, regulation of cell proliferation, cell cycle phase, etc. (Fig. 1C), which are consistent with previous studies^25^,^26^. That is, AFB_1_ exposure mainly affected gene expression of cancer-related and cell cycle-related pathways. Interestingly, GO analysis of DEGs between the AR sample and the Ctrl sample identified enrichment for response to organic substance, response to wounding, negative regulation of proteolysis, etc. without a primary function theme (Fig. 1D). This suggests that intricate regulatory mechanisms involving a variety of pathways contribute to AFB_1_ resistance. Nevertheless, AFB_1_ carcinogenesis and AFB_1_ resistance affect different gene sets with distinct functions.

### AFB_1_ exposure affected lipid catabolism and apoptosis in a different mode

**Figure 2:**
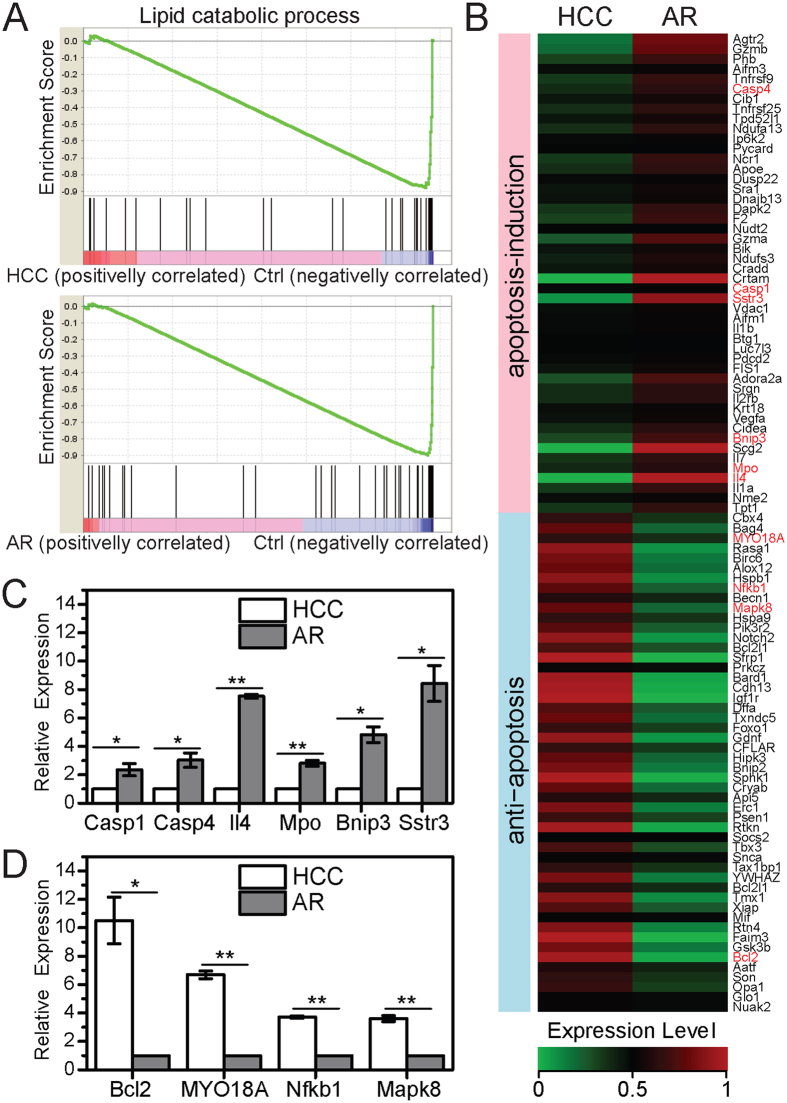
Distinct expression change patterns of genes related to lipid catabolic process and apoptosis in response to AFB_1_ exposure. (**A**) Genes in lipid catabolic process are up-regulated in both the HCC sample and the AR sample compared to the control sample (p-value < 0.001, Enrichment Score = 0.82). (**B**) Anti-apoptosis genes are highly expressed in the HCC sample, whereas apoptosis-induction genes are highly expressed in the AR sample. (**C**) qPCR results confirm significantly increased expression level of selected apoptosis-induction genes in the AR sample (*p-value < 0.05, **p-value < 0.01, Student’s t-test). (**D**) qPCR results confirm significantly increased expression level of selected anti-apoptosis genes in the HCC sample (*p-value < 0.05, **p-value < 0.01, Student’s t-test).

The previous studies showed that lipid peroxidation and apoptosis were two important factors for HCC in response to AFB_1_ exposure^5^,^24^,^25^. However, we don’t know the case in AFB_1_ resistance due to lack of AFB_1_ resistance sample in the previous studies. The AFB_1_ resistance sample in our study allows us to shed light on this. To reach this aim, we applied a powerful tool Gene Set Enrichment Analysis (GSEA)^29^ to gene expression profiles of the three groups of rats. The results showed that genes related to lipid catabolic process were up-regulated in both the HCC and AR samples compared to the Ctrl sample (Fig. 2A). In contrast, apoptosis-related genes were regulated reversely between the HCC sample and the AR sample. The apoptosis-induction genes were up-regulated in the AR sample in response to AFB_1_ exposure but not in the HCC sample (Fig. 2B). Reversely, the anti-apoptosis genes were up-regulated in the HCC sample in response to AFB_1_ exposure, but not in the AR sample. The quantitative analysis of the representative apoptosis-induction and anti-apoptosis genes confirmed this opposite expression change pattern between the HCC and AR sample in response to AFB_1_ exposure (Fig. 2C,D).

### Expression profiles of long non-coding RNAs in response to AFB_1_ exposure

**Figure 3:**
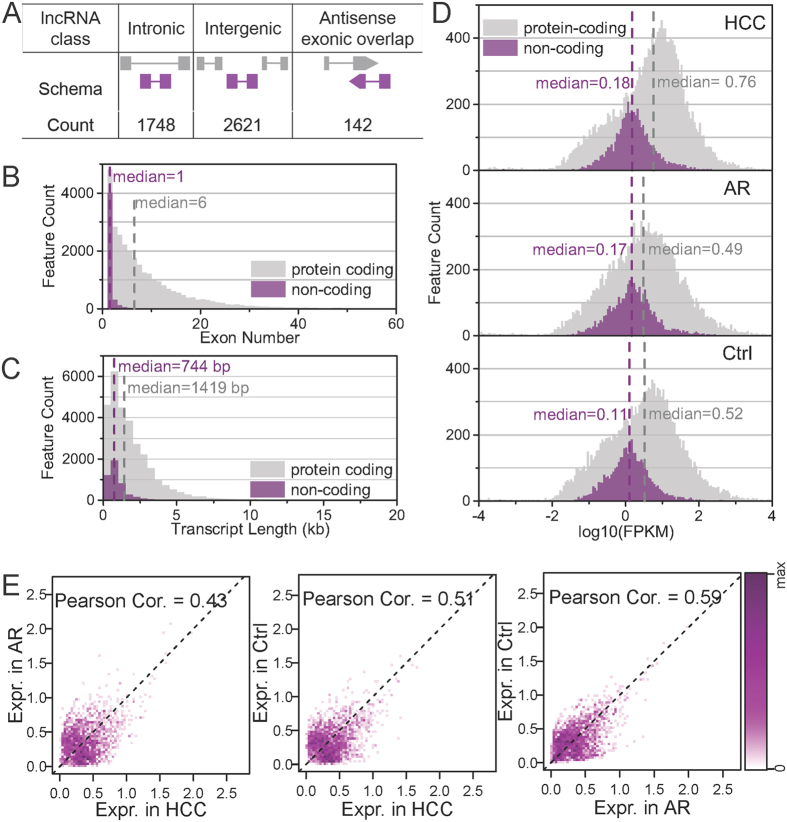
Features of *de novo* lncRNAs and the expression change. (**A**) Schema for the genomic loci of the three category of lncRNAs and their count. (**B**–**C**) lncRNAs have less exons and shorter lengths than protein-coding genes. (**D**) lncRNAs have lower expression levels than protein-coding genes. (**E**) Pairwise correlation of expression levels of lncRNAs between the three samples. The expression levels were measured by log_10_(FPKM+1) and the Pearson correlation coefficients are indicated. Low coefficients indicate extensive alteration in expression by AFB_1_ exposure.

Long non-coding RNAs (lncRNAs) play an important role in regulating gene expression. However, their expression change and functions in response to AFB_1_ exposure remains elusive. To address this issue, we first identified lncRNAs because there were no available well-annotated lncRNAs for rats. We identified a total 4,511 lncRNAs with high confidence (see Methods for details). These lncRNAs were further divided into three groups based on their location in the genome: intergenic, intronic, and antisense exonic (Fig. 3A). Only 3% (142) of them are antisense exonic lncRNAs. The majority (58%) is long intergenic non-coding RNAs (lincRNAs). Comparison analysis found that lncRNAs had fewer exons (about 1.2 per transcript) than protein-coding transcripts (about 8.5) (Fig. 3B). We also found that lncRNAs were shorter than protein-coding transcripts (mean length of 939.5 nt for lncRNAs versus 1855.9 nt for coding transcripts) (Fig. 3C). Moreover, the expression level of lncRNAs were about 16-fold lower on average than that of protein-coding transcripts (Fig. 3D). This indicates that rat lncRNAs have features similar to lncRNAs of other animals^30^,^31^,^32^.

To explore the change in expression of lncRNAs in response to AFB_1_ exposure, we correlated expression profiles of lncRNAs between the three samples. The correlation coefficients are approximately 0.5 (Fig. 3E). The result suggests that AFB_1_ exposure caused extensive alteration in lncRNA expression. The low correlation between the HCC and the AR sample also implies that aflatoxin carcinogenesis and resistance are attributed to gene sets of different functional theme.

### Distinct roles of lncRNAs in aflatoxin carcinogenesis and resistance

**Figure 4:**
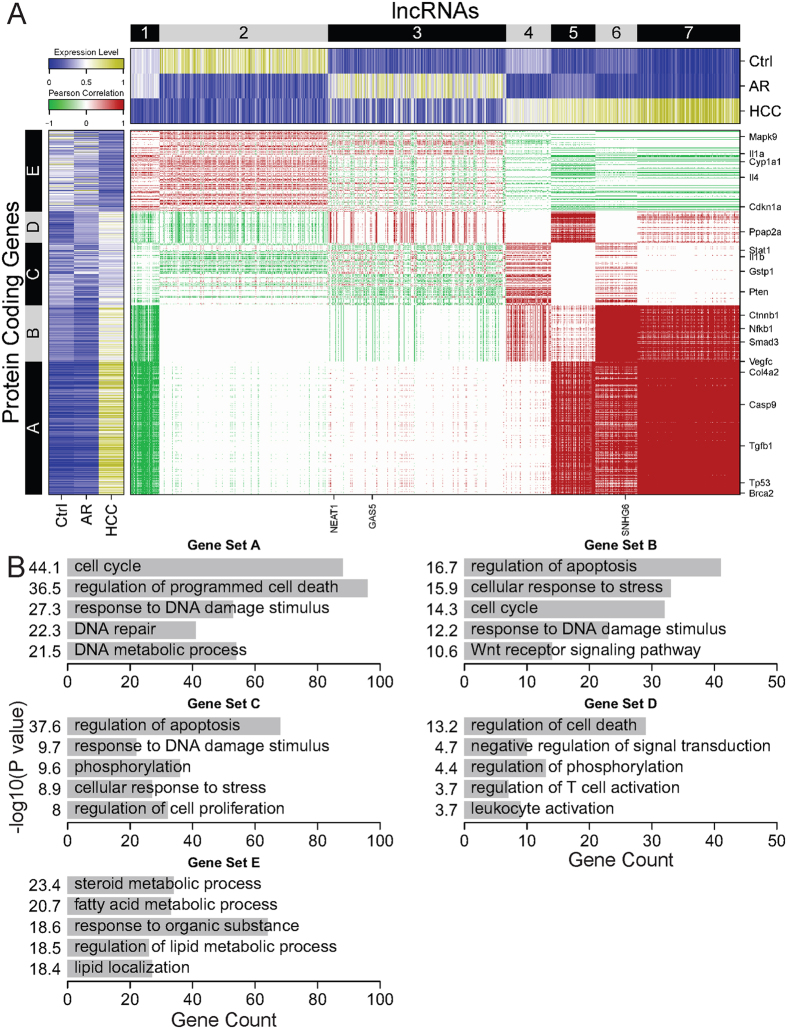
Correlation patterns of expression profiles of protein-coding genes and lncRNAs. (**A**) The heatmap in the central panel shows the expression-based correlation matrix of 4511 lncRNAs (columns) and functional gene sets (rows) derived from gene set enrichment analysis. Red: positive correlation, white: no correlation, green: negative correlation. The heatmap in the top panel shows the expression level of lncRNAs in the three samples. The heatmap in the left panel shows the expression level of protein-coding genes in the three samples. Olive: high, white: intermediate, navy: low. (**B**) Biological process GO terms for which the five gene sets in (**A**) are enriched.

It’s a challenge to investigate the functions of lncRNAs due to the lack of well-annotated features. Therefore, “guilt-by-association” analyses are often applied to predict functions for mammalian lncRNAs^30^,^33^,^34^. Therefore, we correlated expression profiles of protein-coding genes and lncRNAs in the three samples. The correlation matrix between protein-coding genes and lncRNAs were constructed (see Methods for details). We further clustered the matrix and obtained seven lncRNAs clusters and five protein-coding gene sets (Fig. 4A). Almost half of lncRNAs (cluster 4–7) have a higher expression level in the HCC sample than in another two samples. This group of lncRNAs are positively correlated with protein-coding genes (set A&B). Cluster 1 of lncRNAs have an opposite expression pattern. Remarkably, this group of lncRNAs are negatively correlated with the same set of protein-coding genes. Functional analysis of set A protein-coding genes identified enrichment for cell cycle, regulation of programmed cell death, DNA repair (Fig. 4B). GO term analysis also revealed that set B protein-coding genes were enriched for regulation of apoptosis, cell cycle, response to DNA damage stimulus, and Wnt receptor signaling pathway. This finding suggests that a sufficient proportion of lncRNAs contribute to hepatocellular carcinogenesis in response to aflatoxin exposure via up- and down-regulating protein-coding genes of cancer-related functions and signaling.

In contrast, about one fourth of lncRNAs (cluster 3) have a higher expression level in the AR sample than in another two samples. Similarly, another approximately one fourth of lncRNAs (cluster 2) have a higher expression level only in the Ctrl sample (Fig. 4A). These two clusters of lncRNAs have very weak or no correlation with most of set A and B genes, indicating these lncRNAs play no roles in carcinogenesis in response to aflatoxin. Notably, both Ctrl and AR samples did not develop HCC. This suggests that these two clusters of lncRNAs are involved in aflatoxin resistance. The correlation analysis found that they had complex regulatory patterns on the corresponding protein-coding genes (set C-E). GO term analysis of these gene sets showed that they had different functions, such as regulation of phosphorylation, cellular response to stress, regulation of T cell activation, leukocyte activation, steroid metabolic process, fatty acid metabolic process, as well as cancer-related functions (apoptosis, response to DNA damage stimulus) (Fig. 4B). Taken together, lncRNAs contribute in aflatoxin resistance through an intricate regulatory mechanism.

## Discussion

Aflatoxin B_1_ is the most toxic and potent hepatotoxic and hepatocarcinogenic natural compound. Previous studies revealed that AFB_1_ induces mutations in the *TP53* tumor suppressor gene^12^,^13^,^14^, alterations in the expression of protein-coding and miRNA genes^24^,^25^,^26^,^27^, and the synergistic effect on hepatocellular carcinogenesis of AFB_1_ exposure and HBV infection^17^,^18^,^19^,^20^. However, there are no reports about the role of lncRNA in regulating gene expression in response to AFB_1_ exposure. In this study, we identified lncRNAs in rat liver tissues and generated lncRNA expression profiles with and without AFB_1_ treatment. The lncRNAs share the similar features of the counterparts in other animals: few exons, short length, low expression compared to protein-coding genes. AFB_1_ treatment resulted in extensive disturbance in lncRNA expression in liver tissues. Moreover, AFB_1_ exposure regulated different sets of lncRNAs in AFB_1_ carcinogenesis and resistance, respectively. Clustering analysis of the correlation relationship between protein-coding and lncRNA gene expression found that a large group of lncRNAs were highly expressed only in the AFB_1_ hepatocellular carcinogenesis (HCC sample) and up-regulated a large set of protein-coding genes with cancer-related functions such as cell cycle, regulation of programmed cell death, and DNA repair. Intriguingly, these genes were down-regulated by lncRNAs that were highly expressed in AR sample. In contrast, approximately one third of lncRNAs highly expressed only in the AFB_1_ resistance (AR sample) showed a complex pattern of regulating protein-coding genes. These findings suggest that lncRNAs play a more straightforward role in AFB_1_ carcinogenesis through regulating cancer-related genes.

Hepatocellular carcinoma from aflatoxin exposure is a serious global health problem. Although many studies have been conducted on the molecular mechanisms of carcinogenesis from aflatoxin exposure, they all focused on differentially expressed genes in carcinogenesis from aflatoxin exposure. Previous work lacks the molecular mechanism analysis of gene expression change in aflatoxin resistance that may provide more direct cues on reducing the risk for hepatocellular carcinogenesis from aflatoxin exposure. As a matter of fact, our study found that AFB_1_ exposure caused alterations in gene expression (both protein-coding and lncRNA) in AFB_1_ resistance different from AFB_1_ induced hepatocellular carcinogenesis. Anti-apoptosis genes (for example *Bcl2*, *Mapk8*, *Nfkb1*) were up-regulated in the HCC sample whereas apoptosis-induction genes (for example *Casp1*, *Il4*, *Mpo*) were down-regulated in the AR sample. This suggests that apoptosis-related genes play an important role in hepatocellular carcinogenesis in response to AFB_1_ exposure. Therefore, the AFB_1_-resistance sample in our study can facilitate to identify the causal genes or biological processes for hepatocellular carcinogenesis from AFB_1_ exposure by excluding the non-causal ones. These genes can serve as candidate targets for further study to reduce carcinogenesis risk from AFB_1_ exposure.

RNA-seq is a powerful approach to study global gene expression changes. It provides unprecedented coverage of the transcriptome at single nucleotide resolution. Moreover, it can detect gene expression changes at a greater dynamic range and distinguish isoforms. Therefore, RNA-seq provides deeper insights into critical pathways and molecular events in response to AFB_1_ exposure. Our results show that apoptosis plays an important role in both AFB_1_-induced hepatocellular carcinogenesis and AFB_1_ resistance. For example, anti-apoptosis genes *Bcl2*, *Mapk8*, *Nfkb1* were up-regulated in hepatocellular carcinogenesis, whereas apoptosis-induction genes *Casp1*, *Il4*, *Mpo* were down-regulated in AFB_1_ resistance. Collectively, RNA-seq provides an effective tool to investigate toxin-mediated disturbance in the transcriptome.

## Methods

### Ethics statement

All procedures performed in studies involving animals were in accordance with the ethical standards and approved by the Animal Care and Use Committee of The Tenth People’s Hospital of Shanghai (Permit number: 2011-RES1). This study was also approved by Science and Technology Commission of Shanghai Municipality (Approval ID: SYXK 2007-0006).

### Animals and Reagents

Four-week old Wistar male rats (weighing 40 ~ 60 g) were obtained from Animal laboratory center of Guangxi Medical University. Animals were raised individually in stainless steel cages of 30 × 22 × 20 cm^3^, with room temperature of 23 ± 1 °C and relative humidity of 70 ± 10%, with *ad libitum* access to filtered tap water and commercial feed. The animal laboratory rooms were cleaned twice a day and disinfected by UV for one hour once a week.

AFB_1_ (No.A-6636) and DMSO were purchased from Sigma Chemical Co., USA. The pentobarbital sodium (Lot.No.WS69020100) is a production from China Pharmaceutical Group Chemical Reagent Co., Ltd.

### AFB_1_ administration

After the 4-week acclimation period, all rats were randomly divided into control and treated groups. The treated group (60 rats) were administered AFB_1_ by intraperitoneal injection using the following procedure (illustrated in Supplementary Fig. S2): 200 μg/kg, three times a week from the 4^th^–7^th^ week and the 9^th^–12^th^ week; 100 μg/kg, twice a week from the 14^th^–17^th^ week, the 19^th^–22^nd^ week, the 24^th^–27^th^ week, and the 29^th^–32^nd^ week; 100 μg/kg, once a week from the 34^th^–37^th^ week, the 39^th^–42^nd^ week, the 44^th^–47^th^ week, and the 49^th^–52^nd^ week; 100 μg/kg, once a week from the 54^th^–62^nd^ week. Liver biopsy was applied to examine the hepatocarcinoma every 10 weeks from 13^th^ week. 42 rats administered AFB_1_ survived the 70^th^ week when livers were obtained for RNA-seq. 11 rats didn’t develop hepatocellular carcinoma and were grouped as AFB_1_ resistance sample. The remaining 31 rats developed hepatocellular carcinoma and the cancer tissue of livers were collected as the hepatocellular carcinogenesis sample. The 30 rats in the control group without AFB_1_ exposure were also sacrificed to provide liver tissues for RNA-seq at the 70^th^ week. The obtained liver tissues were minced quickly into very small pieces and frozen in liquid nitrogen. All rats were sacrificed after tissue collection by neck dislocation with administration of anesthetic. We also applied humane endpoints (body weight changes, external physical appearance, and behavioral changes) to the experiment rats during the entire study.

### RNA isolation and RNA-seq

Total RNA from 130–150 mg of liver tissue was extracted by TRIzol according to the manufacturer’s protocol (Invitrogen, USA). Reverse transcription was performed using a PrimeScript RT reagent kit (RR037A, Takara Bio Inc, Otsu, Shiga, Japan). The RNA sequencing libraries were constructed from the extracted RNA using standard Illumina libraries prep protocols. RNA-seq was performed on the Illumina HiSeq2000 platform, pair-end sequencing with read length of 90 bp.

### RNA-seq reads mapping

We first used the tool FastQC (http://www.bioinformatics.babraham.ac.uk/projects/fastqc/) to examine sequencing quality and found that no reads needed to be removed. Next, we mapped sequencing reads to rat genome (Rnor_5.0.75) and gene annotations using Tophat2 (v2.0.9)^35^ with up to 5 mismatches. The option “--no-novel-juncs” was turned off as default in order to obtain novel transcripts for *de novo* lncRNA annotation. The genome sequence and gene model annotation were downloaded from Ensembl database (ftp://ftp.ensembl.org/pub/release-75/fasta/rattus_norvegicus/dna/, ftp://ftp.ensembl.org/pub/release-75/gtf/rattus_norvegicus). The read count and mapping results were summarized in Supplementary Table S1.

### *De novo* lncRNA annotation and expression level calculation of lncRNAs

Properly aligned concordant read pairs were retrieved by SAMtools^36^ as input for Cufflinks (v1.3.0) to predict *de novo* transcripts. Default options of Cufflinks were used, except that “-u” was used to weigh multihit reads for accuracy improving. We next employed the tool Cuffcompare to compare the predicted *de novo* transcripts with known rat gene model annotation (Rnor_5.0.75). The transcripts marked by “i”, “u”, “x” were potential non-coding RNAs and represented three different ncRNA categories, namely intronic, intergenic and antisense exonic lncRNAs, respectively. We further filtered the transcripts and retained those with length ≥200 nucleotides, ORF length ≤ 100 amino acids and CPC score > −1. CPC is a support vector machine-based classifier, the coding potential calculator, widely used in non-coding RNA annotation^37^.

### Identification of differentially expressed genes

We merged the *de novo* lncRNA annotation with the known rat gene model annotation (Rnor_5.0.75) to form the full rat gene model annotation as input for the tool Cuffdiff to calculate expression level for each transcript and identify differentially expressed genes (both protein-coding and lncRNA genes) by pairwise comparison of the three samples. The genes whose expression level with fold change > 2 at false discovery rate (FDR) < 0.05 between two samples were defined as differentially expressed genes.

### Correlation analysis of expression level of lncRNA and protein-coding genes

The analysis was conducted in a manner similar to the previous work^34^. Briefly, each lncRNA was associated with a list of protein-coding genes with top correlation coefficients that were further subject to gene set enrichment analysis (GSEA)^29^. Then a correlation matrix between lncRNA and protein-coding genes was constructed, in which each row represents a protein-coding gene and each column represents a lncRNA. Both rows and columns were clustered using k-means. We tried several K values and found that K = 5 for rows and K = 7 for columns gave distinct patterns among clusters and least variation within each cluster. Then the clustering result of the correlation matrix was presented in a heatmap. The expression level of protein-coding genes and lncRNAs were plotted in a heatmap as well.

### Quantitative PCR

The Realtime PCR was carried out in triplicate according to the protocol of the KAPA SYBR FAST Universal qPCR Kit (KK4601, Kapa Biosystems, MA, USA). The relative gene expression levels were calculated using the ∆∆Ct method for relative quantization using 18S or U6 as endogenous reference genes. The primers are provided in Supplementary Table S2.

## Additional Information

**Accession codes:** The RNA-seq data sets have been deposited in Gene Expression Omnibus (GEO) database under accession number GSE70097.

**How to cite this article**: Shi, J. *et al.* Distinct response of the hepatic transcriptome to Aflatoxin B_1_ induced hepatocellular carcinogenesis and resistance in rats. *Sci. Rep.* **6**, 31898; doi: 10.1038/srep31898 (2016).

## Supplementary Material

Supplementary Information
