# Supplementary material for: Distinct response of the hepatic transcriptome to Aflatoxin B1 induced hepatocellular carcinogenesis and resistance in rats
Source: Sci Rep. 2016 Aug 22;6:31898. doi: 10.1038/srep31898 (PMC4992951; doi:10.1038/srep31898)
Supplement: Supplementary Information [file srep31898-s1.pdf]

Distinct response of the hepatic transcriptome to Aflatoxin B_1 induced hepatocellular carcinogenesis and resistance in rats

Jiejun Shi[1,2,+], Jiangtu He[1,+], Jing Lin[2], Xin Sun[2], Fenyong Sun[1,*], Chao Ou[3,*], Cizhong Jiang[1,2,*]

[1] Department of Clinical Laboratory Medicine, Shanghai Tenth People's Hospital of Tongji University, Shanghai, China

[2] Shanghai Key Laboratory of Signaling and Disease Research, the School of Life Sciences and Technology, Tongji University, Shanghai, China

[3] Department of Clinical Laboratory, the Affiliated Tumor Hospital of Guangxi Medical University, Nanning, Guangxi Province, China

*** Corresponding author**

sunfenyong@126.com (F.S.), chaoou_gxmu@163.com (C.O.), or czjiang@tongji.edu.cn (C.J.)

[+]These authors contributed equally to this work.

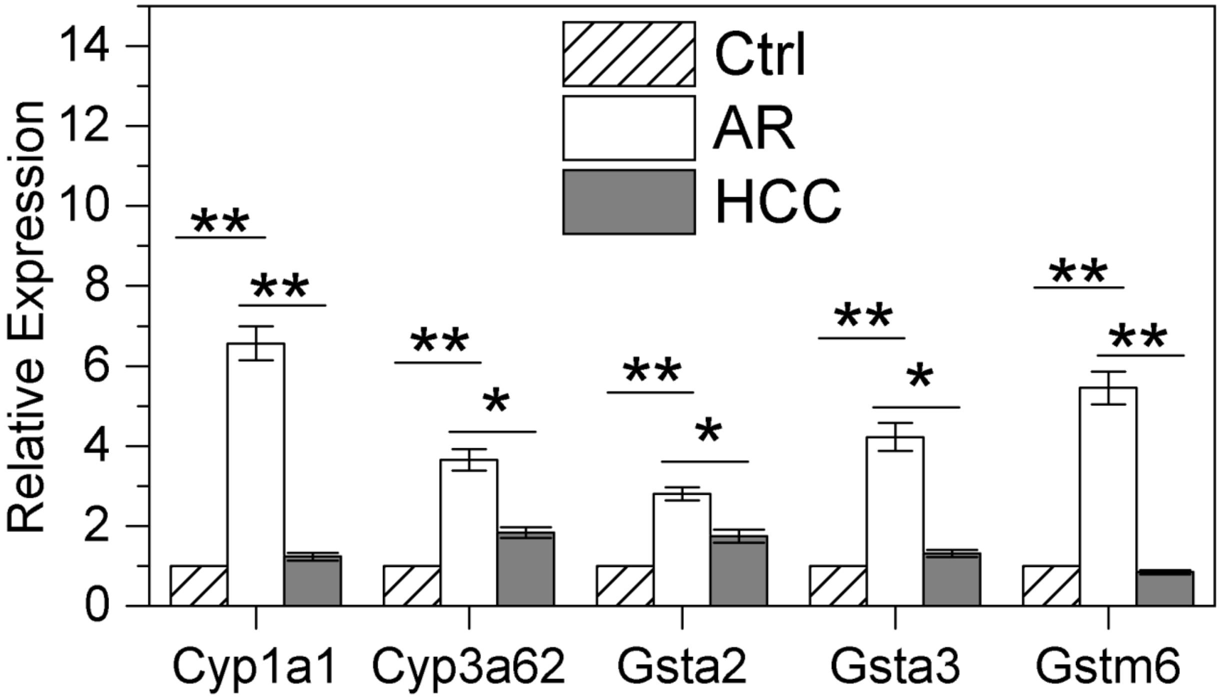

Supplementary Figure S1. qPCR results of AFB1 metabolism related genes.

qPCR results confirm significantly increased expression level of AFB1 metabolism related genes in the AR sample (* p-value < 0.05, ** p-value < 0.01, Student's t-test).

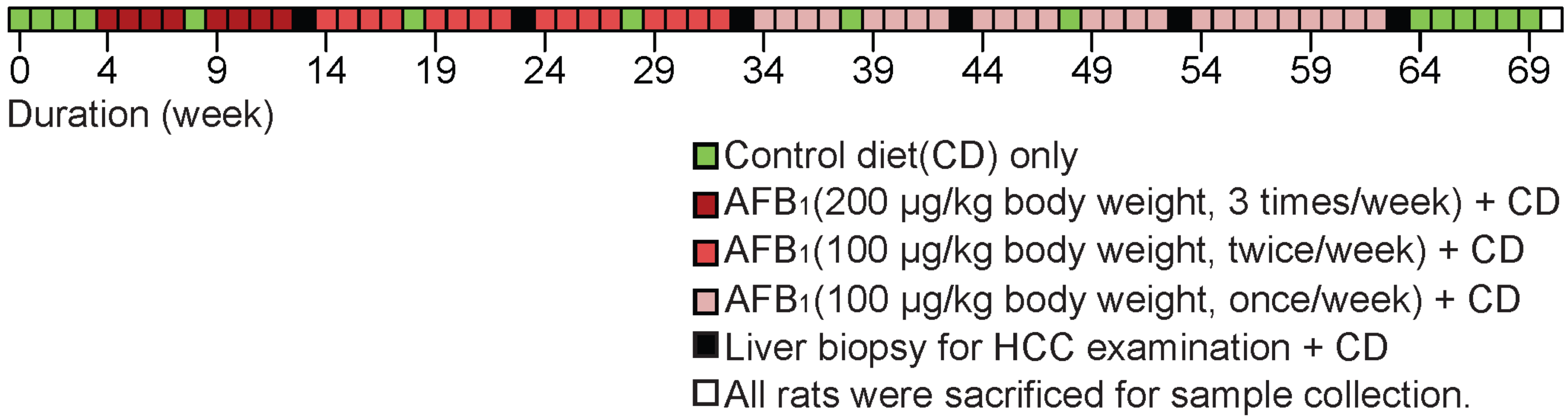

Supplementary Figure S2. AFB1 exposure strategy.

Supplementary Table S1. RNA-seq read count and alignment summary.

Samples	Hepatocellular carcinoma				AFB_1 resistance				Control			
	R1 reads		R2 reads		R1 reads		R2 reads		R1 reads		R2 reads	
	Count	%	Count	%	Count	%	Count	%	Count	%	Count	%
Total reads	29,767,084	100	29,767,084	100	33,333,334	100	33,333,334	100	33,333,334	100	33,333,334	100
Total mapped reads	26,983,873	91	26,995,184	91	30,392,438	91	30,410,670	91	30,144,262	90	30,134,074	90
Mapped concordant paired reads	26,022,103	87	26,022,103	87	29,424,474	88	29,424,474	88	28,993,816	87	28,993,816	87

Supplementary Table S2. Primers used for qPCR.

Gene	Forward primer	Reverse primer
Bcl2	5’-cagagatgtccagtcagctg-3’	5’-cagtcatccacagagcgatg-3’
MYO18A	5’-caagctcagctcgaagagtc-3’	5’-actggccatgctctccagac-3’
Nfkb1	5’-cagctgacagaagacacgag-3’	5’-tctgtgtagcccatctgtcg-3’
Mapk8	5’-gaagacagccatgagtgctg-3’	5’-ctgggtacctactcactggt-3’
Casp1	5’-tgtctcatggtctccaggag-3’	5’-ttctctccacggcatgcctg-3’
Casp4	5’-ggtgttaatgtctcatggcac-3’	5’-gcaggcctgcacaatgatgac-3’
Il4	5’-gacagccctctgagagagatc-3’	5’-ccgtggtgttccttgttgcc-3’
Mpo	5’-agagctcaagcgcctgaatc-3’	5’-ctgagtcattgtaggatcgg-3’
Bnip3	5’-cgtctgacaacttccactag-3’	5’-cactaaatcaggaacaccgc-3’
Sstr3	5’-ttcaggtcagctacctagtc-3’	5’-tgctgcaaactgagcttagc-3’
Cyp1a1	5’-caacaggtcatgctaggatc-3’	5’-ggctttgcaaggacaaggag-3’
Cyp3a62	5’-actgatgtggagattgtggc-3’	5’-caggacatcataggtcacag-3’
Gsta2	5’-aaggacatgaaggagagagc-3’	5’-acaaggtagtcttggccatg-3’
Gsta3	5’-gttggcaacaagctgagcag-3’	5’-ttgctgactctggttctcag-3’
Gstm6	5’-cagtggctggatgacaaattc-3’	5’-atgtccacacgaatcctctc-3’
